# Acute acalculous cholecystitis on a COVID-19 patient: a case report

**DOI:** 10.1016/j.amsu.2020.08.027

**Published:** 2020-08-31

**Authors:** E. Mattone, M. Sofia, E. Schembari, V. Palumbo, R. Bonaccorso, V. Randazzo, G. La Greca, C. Iacobello, D. Russello, S. Latteri

**Affiliations:** a Department of Medical Surgical Sciences and Advanced Technologies “Ingrassia”, University of Catania, General Surgery Unit, Cannizzaro Hospital, Via Messina 829, 95126, Catania, Italy; b Infectious Disease Unit, Cannizzaro Hospital, Via Messina 829, 95126, Catania, Italy

**Keywords:** COVID-19 pandemic, Acute acalculous cholecystitis, Cholecystostomy, Laparoscopy, Emergency surgery, Case report

## Abstract

**Introduction:**

We report an extremely rare case of acute acalculous cholecystitis on a COVID-19 patient. In our knowledge, this is the first report of laparoscopic cholecystectomy performed on a COVID-19 patient.

**Presentation of case:**

A COVID-19 patient was diagnosed with acute acalculous cholecystitis and a multidisciplinary team decided to perform a percutaneous transhepatic biliary drainage (PTBD) as the first treatment. SARS-CoV-2 RNA was not found in the bile fluid. Because of deterioration of the patient's clinical conditions, laparoscopic cholecystectomy had to be performed and since the gallbladder was gangrenous, the severe inflammation made surgery difficult to perform.

**Discussion:**

Acalculous cholecystitis was related with mechanical ventilation and prolonged total parenteral nutrition, in this case the gangrenous histopathology pattern and the gallbladder wall ischemia was probably caused by vascular insufficiency secondary to severe acute respiratory distress syndrome of COVID-19 pneumonia. The percutaneous transhepatic gallbladder drainage (PTBD) was performed according to Tokyo Guidelines because of high surgical risk. Laparoscopic cholecystectomy was next performed due to no clinical improvement. The absence of viral RNA in the bile highlights that SARS-CoV-2 is not eliminated with the bile while it probably infects small intestinal enterocytes which is responsible of gastrointestinal symptoms such as anorexia, nausea, vomiting, and diarrhoea.

**Conclusions:**

Although the lack of evidence and guidelines about the management of patient with acute cholecystitis during COVID-19 pandemic, laparoscopic cholecystectomy, at most preceded by PTGBD on high surgical risk patients, remains the gold standard for the treatment of acute cholecystitis on COVID-19 patients.

## Highlights

• Coronavirus disease-19 (COVID-19) has spread to several countries around the world and has become an unprecedented pandemic.
• Cholecystitis was probably caused by vascular insufficiency secondary to respiratory distress syndrome of COVID-19 pneumonia.
• SARS-CoV-2 probably infects small intestinal enterocytes which is responsible of gastrointestinal symptoms such as diarrhoea.
• Laparoscopic cholecystectomy, remains the gold standard for the treatment of acute cholecystitis on COVID-19 patient.

## 1 Introduction

SARS-CoV-2 is a novel coronavirus that has not been previously identified in humans and it's responsible of coronavirus disease-19 (COVID-19) which has spread to several countries around the world and has become an unprecedented pandemic [1]. Infected patients have been reported with common clinical symptoms such as fever, cough, myalgia, dyspnoea and normal or decreased leukocyte counts [2]. COVID-19 patients have been shown to undergo severe acute respiratory distress syndrome, caused by cytokine storm and this is the foremost reason for morbidity and mortality due to multiple organ failure [3]. Although many patients reported gastrointestinal symptoms such as anorexia, nausea, vomiting and diarrhoea, there was no evidence about the involvement of gallbladder and biliary tract in literature to date. We report an extremely rare case of acute acalculous cholecystitis on a COVID-19 patient. In our knowledge, this is the ﬁrst report of laparoscopic cholecystectomy performed on a COVID-19 patient. The paper has been reported in line with the SCARE 2018 criteria [4].

## 2 Case report

A 66-year-old man, ex-smokers since 2006 with no drug and inheritable genetic diseases history, was admitted to our hospital with fever (38,2 °C), non-productive cough, and dyspnoea started by 10-days, after a holiday cruise. The clinical parameters were: 95/min. heart rate, 135/90 mmHg blood arterial pressure and 87%O2 saturation. Respiratory rate was 35/min. breaths, vocal fremitus was diminished bilaterally, especially at the base of the lung, with dullness on percussion and rhonchi, wheezes and rubs at auscultation. The physical examination of the abdomen was negative. The laboratory findings revealed normal white blood cell (WBC) 5.80 K/aeL and high C-reactive protein (CRP) level, 8.20 mg/dL. Chest X-ray showed interstitial lung disease and nasopharyngeal swab-PCR was positive for SARS-CoV-2 infection. The patient was diagnosed with COVID-19 pneumonia and he has been intubated because of respiratory distress. COVID-19 infection was treated in intensive care unit by hydroxychloroquine 200 mg., azithromycin 500 mg., tocilizumab 60 mg., methylprednisolone 70 mg. and enoxaparin 4.000 U.I. This therapeutic protocol led good clinical result, as shown by chest X-rays that reported a decrease of interstitial lung disease after 34-days of hospital stay. At 49th day of hospitalization patient complained right upper quadrant abdominal pain together with nausea, vomiting and mild corporeal temperature (38 °C). The physical examination revealed a tender abdomen with normal peristaltic bowel movements, no jaundice and a positive Murphy's sign. Blood tests showed mild leucocytosis, 11.90 K/aeL, with a shift to the left of neutrophils, 80.80%, and high CRP level, 6.60 mg/dL. The abdominal computed tomography (CT) revealed a severe gallbladder distension, pericholecystic fluid, marked gallbladder wall thickening, no gallstones were identified and the extrahepatic bile ducts were normal. The patient was diagnosed with acute acalculous cholecystitis and a multidisciplinary team including general surgery unit, interventional radiology unit, intensive care unit and infectious disease unit, had discussed the therapeutic choices for patient.

A percutaneous transhepatic biliary drainage (PTBD) under ultrasound control of gallbladder, was performed as the first. A sample of bile was tested for SARS-CoV-2 RNA and it was negative. After 3-days there was no clinical improvement with persistent high WBC count, 13.50 K/aeL. The multidisciplinary team met and a laparoscopic surgical procedure was planned. Laparoscopic cholecystectomy was made by the chief of general surgery helped by a consultant surgeon and it was performed through four ports. The gallbladder was gangrenous and a severe inflammation make the Calot's elements diﬃcult to identify. However, the cystic duct and the cystic artery were eventually identified and dissected. Therefore, cholecystectomy was finished. The operative time was 105 minutes. On the 3rd post-operative day nasopharyngeal swab-PCR became negative for SARS-CoV-2 RNA and further nasopharyngeal swab-PCR performed after 48 hours and after 96 hours were negative too. The postoperative course was uneventful and it was well-tolerated by the patient, so he was discharged on the 9th postoperative day in satisfactory condition.

## 3 Discussion

Acalculous cholecystitis is an uncommon disease caused by hypomotility of the gallbladder which is responsible of increased intraluminal pressures evolving in inflammation, ischemia and necrosis [5]. It accounts for only 10% of acute cholecystitis but it has higher morbidity and mortality than calculous cholecystitis [6,7]. Acalculous cholecystitis is also strictly related with other pathological condition such as trauma, cardiopulmonary resuscitation, mechanical ventilation, sepsis, burn, prolonged total parenteral nutrition and major surgery [5]. In addition, Croteau et al. reported seven cases of acute acalculous cholecystitis occurred in patients with relapsing - remitting multiple sclerosis during or shortly after alemtuzumab treatment, suggesting an acute cytokine release syndrome as a pathogenic mechanism [8]. Gangrenous cholecystitis is caused by ischemia arising from vascular insufficiency and it evolves into necrosis and perforation of the gallbladder wall with bile leakage [9]. Surgical societies released their recommendations to manage surgical disease during the COVID-19 pandemic [10]. The recommendations on emergency surgery have fuelled a debate among surgeons on an international level [10]. Reports from China showed that asymptomatic COVID-19-positive patients underwent surgery presented poor clinical outcomes, with an increasing of mortality and pulmonary complication rate [11]. In the present case acalculous cholecystitis was related with mechanical ventilation and prolonged total parenteral nutrition, instead the gangrenous histopathology pattern was probably caused by severe acute respiratory distress syndrome of COVID-19 pneumonia which determined vascular insufficiency, responsible of gallbladder wall ischemia. Although laparoscopic cholecystectomy is considered the gold standard for the treatment of most gallbladder disease [12], Tokyo Guidelines advice to delay surgical operation and to perform the percutaneous gallbladder drainage for surgically high-risk patients with acute cholecystitis and comorbidities [13]. Multidisciplinary team decided to perform surgery after cholecystostomy due to worsening clinical condition of the patient, presenting fever and right upper quadrant abdominal pain and patient agreed with doctors because of his poor clinical conditions. Laparoscopic cholecystectomy was preferred to open cholecystectomy because of the lack of evidence about an increased risk of SARS-CoV-2 infection during laparoscopy [10]. Finally, SARS-CoV-2 RNA was detected from a variety respiratory sources such as throat, nasal nasopharyngeal, sputum and bronchoalveolar lavage fluid, but it was also detected in faeces [14], placental and foetal membrane [15]. In our acknowledgement this is the first case of attempt at trying to demonstrate the presence of viral RNA in the bile but the result was negative and this highlights that SARS-CoV-2 is not eliminated with the bile while it probably infects small intestinal enterocytes [16] which is responsible of gastrointestinal symptoms such as anorexia, nausea, vomiting, and diarrhoea [17].

## 4 Conclusions

The lack of evidence and guidelines about the management of patient with acute cholecystitis during COVID-19 pandemic lead surgeons from the better decision for patient following the same guidelines used for the assessment and the treatment of COVID-19 negative patient. Therefore, laparoscopic cholecystectomy, preceded by percutaneous transhepatic gallbladder drainage (PTBD) in high-risk patient for surgical treatment, remain the best choice and the gold standard for the treatment of acute cholecystitis on COVID-19 patient.

## Consent statement

Written informed consent was obtained from the patient for publication of this case report and accompanying images. A copy of the written consent is available for review by the Editor-in-Chief of this journal on request.

## Provenance and peer review

Not commissioned, externally peer reviewed.

## Sources of funding

This research did not receive any grant from funding agencies in the References public, commercial, or not-for-profit sectors.

## Ethical approval

Not applicable.

## Consent

The patient signed informed consent and written permission to use data for scientific purposes.

## Author contribution

Mattone Edoardo: writing the paper.

Sofia Maria: data analysis.

Schembari Elena: data collection.

Palumbo Valentina: data collection.

Bonaccorso Rosario: data interpretation.

Randazzo Valentina: data analysis La Greca Gaetano: data interpretation.

Iacobello Carmelo: data interpretation.

Russello Domenico: study concept.

Latteri Saverio: study concept and design.

## Guarantor

Russello Domenico: russello@unict.it.

## Declaration of competing interest

All authors of the manuscript have read and agreed to its content and are accountable for all aspects of the accuracy and integrity of the manuscript in accordance with ICMJE criteria. The article is original, has not already been published in a journal, and is not currently under consideration by another journal. All authors agree to the terms of the BioMed Central Copyright and License Agreement and Open Data policy.

## List of abbreviations

PTBD: Percutaneous transhepatic biliary drainage
COVID-19: Coronavirus disease -19

## References

[1] Saxena S.K. (2020). Series: Medical Virology: from Pathogenesis to Disease Control, Coronavirus Disease 2019 (COVID-19).

[2] Zhang J.J., Dong X., Cao Y.Y., Yuan Y.D., Yang Y.B., Yan Y.Q., Akdis C.A., Gao Y.D. (2020). Clinical characteristics of 140 patients infected with SARS-CoV-2 in Wuhan, China. Allergy.

[3] Zhao D., Yao F., Wang L., Zheng L., Gao Y., Ye J., Guo F., Zhao H., Gao R. (2020). A comparative study on the clinical features of COVID-19 pneumonia to other pneumonias. Clin. Infect. Dis..

[4] Agha R.A., Borrelli M.R., Farwana R., Koshy K., Fowler A., Orgill D.P., For the Scare Group (2018). The SCARE 2018 statement: updating consensus surgical CAse REport (SCARE) guidelines. Int. J. Surg..

[5] Balmadrid B. (2018). Recent advances in management of acalculous cholecystitis [version 1; referees: 2 approved]. F1000Research.

[6] Barie P.S., Fischer E. (1995). Acute acalculous cholecystitis. J. Am. Coll. Surg..

[7] Pesce A., La Greca G., Latteri S. (2017 Apr). Laparo-endoscopic rendez-vous versus sequential "delayed" approach in patients with CholedocholithiasisClinical trial minerva. Chir.

[8] Croteau D., Flowers C., Kulick C.G. (2018). Acute acalculous cholecystitis: a new safety risk for patients with MS treated with alemtuzumab. Neurology published online March.

[9] Katabathina V.S., Zafar A.M., Suri R. (2015 Dec 1). Clinical presentation, imaging, and management of acute cholecystitis. Tech. Vasc. Intervent. Radiol..

[10] Campanile F.C., Podda M., Arezzo A. (2020). Acute cholecystitis during COVID-19 pandemic: a multisocietary position statement. World J. Emerg. Surg..

[11] Lei S., Jiang F., Su W. (2020). Clinical characteristics and outcomes of patients undergoing surgeries during the incubation period of COVID-19 infection. Eclin Med.

[12] Mattone E., Latteri S., Teodor T. (2018 Mar). Dystopic retrohepatic gallbladder and cholecysto‐choledocho lithiasis: the rendez‐vous and indocyanine green fluorescence. Clin Case Rep.

[13] Mori Y., Ito T., Baron T.H. (2018). Tokyo guidelines 2018: management strategies for gallbladder drainage in patients with acute cholecystitis (with videos). J Hepatobiliary Pancreat Sci.

[14] Loeffelholz M.J., Tang Y.W. (2020 9). Laboratory diagnosis of emerging human coronavirus infections – the state of the art. Emerg. Microb. Infect..

[15] Penfield C.A., Brubaker S.G., Limaye L.A. (2020). Detection of severe acute respiratory syndrome coronavirus 2 in placental and fetal membrane samples. 5Am J Obstet Gynecol MFM..

[16] Mönkemüller K., Fry L., Rickes S. (2020). COVID-19, coronavirus, SARS-CoV-2 and the small bowel. Rev. Esp. Enferm. Dig..

[17] Agarwal A., Chen A., Ravindran N., To C., Thuluvath P.J. (2020). Gastrointestinal and liver manifestations of COVID-19. J Clin Exp Hepatol.

